# IL-10-producing regulatory B cells (B10 cells) in autoimmune disease

**DOI:** 10.1186/ar3907

**Published:** 2013-02-11

**Authors:** Ioannis Kalampokis, Ayumi Yoshizaki, Thomas F Tedder

**Affiliations:** 1 Box 3010, Department of Immunology, Room 353 Jones Building, Research Drive, Duke University Medical Center, Durham, NC 27710, USA

## Abstract

B cell abnormalities contribute to the development and progress of autoimmune disease. Traditionally, the role of B cells in autoimmune disease was thought to be predominantly limited to the production of autoantibodies. Nevertheless, in addition to autoantibody production, B cells have other functions potentially relevant to autoimmunity. Such functions include antigen presentation to and activation of T cells, expression of co-stimulatory molecules and cytokine production. Recently, the ability of B cells to negatively regulate cellular immune responses and inflammation has been described and the concept of regulatory B cells has emerged. A variety of cytokines produced by regulatory B cell subsets have been reported, with IL-10 being the most studied. In this review, this specific IL-10-producing subset of regulatory B cells has been labeled B10 cells to highlight that the regulatory function of these rare B cells is mediated by IL-10, and to distinguish them from other B cell subsets that regulate immune responses through different mechanisms. B10 cells are a functionally defined subset currently identified only by their competency to produce and secrete IL-10 following appropriate stimulation. Although B10 cells share surface markers with other previously defined B cell subsets, currently there is no cell surface or intracellular phenotypic marker or set of markers unique to B10 cells. The recent discovery of an effective way to expand B10 cells *ex vivo *opens new horizons in the potential therapeutic applications of this rare B cell subset. This review highlights the current knowledge on B10 cells and discusses their potential as novel therapeutic agents in autoimmunity.

## Introduction

Traditionally, B cells have been thought to contribute to the pathogenesis of autoimmune disease through antigen (Ag)-specfic autoantibody production [1]. Nonetheless, the role of B cells in autoimmunity extends beyond the production of autoantibodies. B cells are now well established to have both positive and negative regulatory roles during immune responses.

B cells can positively regulate immune responses by producing Ag-specfic antibody and inducing optimal T cell activation [2,3]. B cells can serve as professional Ag-presenting cells, capable of presenting Ag 10^3^-fold to 10^4^-fold more efficiently than nonprofessional Ag-presenting cells [4]. B cell Ag presentation is required for optimal Ag-specific CD4^+ ^T cell expansion, memory formation, and cytokine production [5-7]. B cells may also positively regulate CD8^+ ^T cell responses in mouse models of autoimmune disease [8,9]. Furthermore, costimulatory molecules (such as CD80, CD86, and OX40L) expressed on the surface of B cells are required for optimal T cell activation [10,11]. The positive regulatory roles of B cells extend to multiple immune system components; the absence of B cells during mouse development results in significant quantitative and qualitative abnormalities within the immune system, including a remarkable decrease in thymocyte numbers and diversity [12], significant defects within spleen dendritic cell and T cell compartments [13-15], absence of Peyer's patch organogenesis and follicular dendritic cell networks [16,17], and absence of marginal zone and metallophilic macrophages with decreased chemokine expression [15,17]. B cells also positively regulate lymphoid tissue organization [18,19]. Finally, dendritic cell, macrophage, and T_H _cell development may all be influenced by B cells during the formation of immune responses [20].

B cells can also negatively regulate cellular immune responses through their production of immunomodulatory cytokines. B cell-negative regulation of immune responses has been demonstrated in a variety of mouse models of autoimmunity and inflammation [21-30]. Although the identification of B cell subsets with negative regulatory functions and the definition of their mechanisms of action are recent events, the important negative regulatory roles of B cells in immune responses are now broadly recognized [31,32]. A variety of regulatory B cell subsets have been described; IL-10-producing regulatory B cells (B10 cells) are the most widely studied regulatory B cell subset [30,31,33]. Comprehensive reviews summarizing the variety of regulatory B cell subsets have been published during recent years [31,32]. The present review will therefore focus exclusively on the IL-10 producing regulatory B cell subset. This specific subset of regulatory B cells has been labeled B10 cells to highlight that the regulatory function of these rare B cells is mediated by IL-10, and to distinguish them from other B cell subsets that regulate immune responses through different mechanisms [34]. This functional subset of B cells is defined solely by its IL-10-dependent regulatory properties and extends beyond the concept of transcription factor-defined cell lineages. This review highlights our current knowledge on B10 cells, with emphasis on their roles in autoimmune disease, and discusses their potential as a novel therapeutic approach in the treatment of autoimmunity.

## Biology of B10 cells

**Figure 1:**
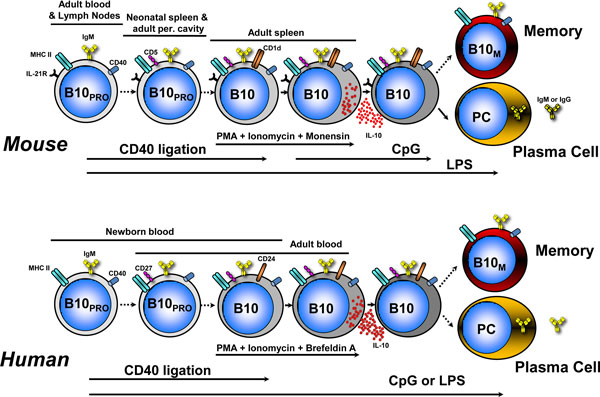
**Linear differentiation model of B10 cell development *in vivo *in mice and humans**. B10 cells originate from a progenitor population (B10_PRO_). In mice, B10_PRO _cells are found in the CD1d^−^CD5^− ^adult blood and lymph node B cell subsets and within the CD1d^−^CD5^+ ^neonatal spleen and adult peritoneal cavity B cell subsets. CD40 stimulation induces B10_PRO _cells to become competent for IL-10 expression, while lipopolysaccharide (LPS) induces B10_PRO _cells to become competent for IL-10 expression and induces B10 cells to produce and secrete IL-10. CD1d^hi^CD5^+ ^IL-10-competent B10 cells in the adult spleen are induced to express IL-10 following stimulation with phorbol esters (phorbol-12-myristate-13-acetate (PMA)) and ionomycin or LPS plus PMA and ionomycin for 5 hours. Following a transient period of IL-10 expression, a small subset of B10 cells can differentiate into antibody-secreting plasma cells (PC). B10 cells also possibly differentiate into memory B10 cells (B10_M_). B10 cell development in humans appears to follow the differentiation scheme observed in mice. B10 cells and B10_PRO _cells have been identified in human newborn and adult blood. B10+B10_PRO _cells in adult human blood express CD27 and CD24. Whether human B10 cells further differentiate into PCs or B10_M _remains to be determined. Solid arrows, known associations; dashed arrows, speculated associations. MHC-II, major histocompatibility complex class II.

One of the most fundamental basic biology questions about B10 cells relates to the stimuli driving their development. Ag and B cell receptor (BCR) signaling are critical in early development, although additional stimuli such as CD40 ligation and Toll-like receptor (TLR) ligands appear to be involved in the developmental process. Figure 1 illustrates our current understanding of B10 cell development *in vivo *both in mice and humans, where their development shows multiple similarities.

B10 cells are a functionally defined B cell subset. There are no unique phenotypic markers for B10 cells, and these cells are currently defined only by their competency to produce and secrete IL-10 following appropriate stimulation. B10 cells share surface markers with other previously defined B cell subsets both in mice and humans, such as marginal zone B cells, transitional B cells, B1a B cells, and memory B cells. However, no one marker or set of markers is unique to B10 cells. For identification of B10 cells, intracellular cytoplasmic IL-10 staining is used, following *ex vivo *stimulation with lipopolysaccharide (LPS) or CpG oligonucleotides, phorbol esters (phorbol-12-myristate-13-acetate (PMA)) and ionomycin for 5 hours [35]. B10 cells originate from a progenitor population (B10_PRO _cells). B10_PRO _cells develop into B10 cells after maturation through CD40 ligation or exposure to LPS or CpG. B10_PRO _cells can be identified indirectly following *ex vivo *stimulation with LPS or CpG in the presence of CD40 ligation for 48 hours with the addition of PMA and ionomycin for the last 5 hours. The IL-10^+ ^B cells measured following this 48-hour stimulation include cells that would have been IL-10^+ ^even with the shorter 5-hour stimulation (B10 cells), and thereby represent the sum of B10 plus B10_PRO _cells (B10+ B10_PRO_).

### Mouse B10 cell development

BCR specificity, affinity and signaling are the most important currently identified factors in B10 cell development. B10 cell regulation of inflammation and autoimmunity is Ag specific [23,30,36]. The importance of BCR diversity is demonstrated by the fact that B10+ B10_PRO _cells are reduced by approximately 90% in transgenic mice with a fixed BCR [37]. Signaling through the BCR appears critical during early development *in vivo*. CD19-deficient mice (where BCR signaling is decreased) have a 70 to 80% decrease in B10+B10_PRO _cells [30]. In contrast, B10 cells are expanded in human CD19 transgenic mice (where the overexpression of CD19 augments BCR signaling). The absence of CD22, which normally dampens CD19 and BCR signaling [38], also results in increased B10 cell numbers. Ectopic B cell expression of CD40L (CD154) in transgenic mice, which induces increased CD40 signaling [39], also increases B10 cell numbers. CD22^−*/*− ^mice that also ectopically express CD40L show dramatically enhanced numbers of CD1d^hi^CD5^+ ^B cells and B10 cells [40]. The induction of IL-10^+ ^B cells with regulatory activity by T cell immunoglobulin domain and mucin domain protein 1 (TIM-1) ligation [41] further highlights the importance of BCR signaling in B10 cell development. BCR signaling and TIM-1 are closely related. BCR ligation induces TIM-1 expression on B cells [41,42], and TIM-1 ligation appears to enhance BCR signaling since it increases antibody production both *in vitro *and *in vivo *[43]. The importance of BCR-related signals is further highlighted by the observation that the stromal interaction molecules 1 (STIM1) and 2 (STIM2) are required for B cell IL-10 production [44]. Remarkably, B cells lacking both stromal interaction molecule proteins failed to produce IL-10 after BCR stimulation in the presence of PMA and ionomycin for 5 hours [44]. All of the above indicate that BCR-related signals are particularly important in B10 cell development.

Despite the requirement for BCR expression and function during mouse B10 cell development, B cell stimulation with mitogenic anti-IgM antibody alone does not induce cytoplasmic IL-10 expression. The combination of anti-IgM stimulation with CD40 ligation and LPS or CpG significantly reduces IL-10 competence [37]. BCR-generated signals thus inhibit the abilities of LPS or CpG and CD40 ligation to induce cytoplasmic IL-10 production. Whether BCR stimulation inhibits the induction of IL-10 competence by inducing B cells to mature or differentiate down a divergent pathway or diverts intracellular signaling is unknown. Another possibility is that the signals generated by mitogenic anti-IgM BCR cross-linking are too intense and that low-affinity Ag--BCR interactions drive B10_PRO _cell development *in vivo*.

A recent study revealed the importance of IL-21, major histocompatibility complex class II (MHC-II) and CD40 during cognate interactions with CD4^+ ^T cells in B10 cell development [36]. *Ex vivo *stimulation of purified spleen CD19^+ ^B cells with IL-21 induced 2.7-fold to 3.2-fold higher B10 cell frequencies, and 4.4-fold to 5.3-fold more IL-10 secretion compared with stimulation with media alone. Remarkably, IL-21 induced B10 cells to produce IL-10 without the need for stimulation with phorbol esters and ionomycin. Interestingly, IL-21 induced a threefold increase in IL-10^+ ^B cells within the splenic CD1d^hi^CD5^+ ^B cell subset, but did not induce IL-10^+ ^B cells within the CD1d^lo^CD5^-- ^B cell subset. Both B10 cells and non-B10 cells expressed IL-21R at similar levels, and *ex vivo *B10, B10pro and CD1d^hi^CD5^+ ^B cell numbers were similar among IL-21R-deficient (IL-21R^--/--^), MHC-II-deficient (MHC-II^--/--^) and CD40-deficient (CD40^--/--^) mice. Nevertheless, IL-21R, MHC-II and CD40 appear to be required for B10 cell effector functions, at least in experimental autoimmune encephalomyelitis (EAE) [36]. Regulatory B10 cell function therefore requires IL-21R signaling, as well as CD40 and MHC-II interactions, potentially explaining Ag-specific B10 cell effector function [37].

Although cognate interactions with CD4^+ ^T cells are important for B10 cell effector functions [36], T cells do not appear to be required for B10 cell development. B10 cells are present in T cell-deficient nude mice, and their frequencies and numbers are approximately fivefold higher when compared with wildtype mice. This observation is strengthened by the fact that MHC class I and MHC-II molecules and CD1d expression are not required for B10 cell development [37]. The presence or absence of T cells *in vitro *also does not affect the frequency of B10 cells. Although increased B10 cell frequencies in T cell-deficient mice suggest that T cells might actually inhibit B10 cell development, it is equally possible that the immunodeficient state of these mice allows subclinical inflammation that induces B10 cell generation. The role of T cells in B10 cell development *in vivo *is thereby complex and, although T cells are not required for B10 cell development, cognate interactions between CD4^+ ^T cells and B10 cells are required for B10 cell effector function.

B10 cells can be driven to produce IL-10 by TLR4 (LPS) or TLR9 (CpG oligonucleotides) ligands. Mouse B10_PRO _cells acquire the ability to function like B10 cells after *in vitro *maturation following stimulation with LPS, but not CpG, in the presence or absence of agonistic CD40 mAb [32]. TLR4 and TLR9 signaling through myeloid differentiation primary response gene 88 (MyD88) is necessary for the optimal maturation and IL-10 induction of B10pro and B10 cells following LPS stimulation and LPS or CpG stimulation, respectively [37]. Nevertheless, MyD88 expression is not an absolute requirement for B10 cell development *in vivo*, since B10 cells develop normally in MyD88^--/-- ^mice [37]. Specifically, numbers of B cells with the capacity to produce IL-10 are equivalent in wildtype and MyD88^--/-- ^mice when their maturation or IL-10 production are measured following CD40 ligation or PMA plus ionomycin stimulation, respectively, demonstrating that B10 _PRO _and B10 cells are present at normal frequencies in MyD88^--/-- ^mice. Thereby, while TLR signaling is not required for B10 cell development, MyD88 expression is required for LPS to induce optimal B cell IL-10 expression and secretion *in vitro*.

The involvement of TLR signals in B10-cell IL-10 production was recently demonstrated [45]. IL-10 production by B cells, stimulated by contact with apoptotic cells, results from the engagement of TLR9 within the B cell after recognition of DNA-containing complexes on the surface of apoptotic cells by the BCR. An earlier study also highlights the effects of apoptotic cells on B cell IL-10 production, where apoptotic cells protected mice from developing collagen-induced arthritis (CIA) by the induction of IL-10-producing regulatory B cells [46]. Cell death products may therefore represent one of the physiologic triggers for B10 cell development by providing a combination of BCR and TLR signals. Additional non-TLR/non-BCR signals (such as alarmins) released from dying cells may be also involved but their identities remain to be determined.

Although certain transcription factors are involved at some point in B10 cell development, it is important to stress that there is no known transcription factor signature unique to B10 cells. Following a transient period of IL-10 transcription characterized by increased expression of the *blimp1 *and *irf4 *transcription factors along with decreased expression of *pax5 *and *bcl6*, a significant but small fraction of B10 cells can differentiate into antibody-secreting cells producing IgM and IgG polyreactive antibodies that are enriched for autoreactivity to single-stranded or double-stranded DNA and histones [47]. Whether B10 cells can produce and secrete IL-10 repeatedly remains to be determined.

### Human B10 cell development

B10_PRO _cells and B10 cells have been recently identified in humans [48] and their responses to LPS, CpG and CD40 ligation appear to follow the general scheme of mouse B10 cell development (Figure 1). One notable difference in mouse versus human B10 cell development is the lack of response of mouse B10_PRO _cells to CpG compared with their human counterparts. Human B10_PRO _cells can be driven to develop *ex vivo *into B10 cells with LPS or CpG stimulation, or CD40 ligation. Interestingly, BCR ligation augmented human B cell IL-10 responses to CpG in one study [49]. This finding is in discordance with our findings in both humans [48] and mice [37], where BCR-generated signals inhibit the abilities of LPS or CpG and CD40 ligation to induce cytoplasmic IL-10 production. Whether human B10 cells develop into antibody-secreting cells or enter the memory pool (memory B10 cells, B10_M_) remains to be determined.

### Unsolved questions on B10 cell development

**Figure 2:**
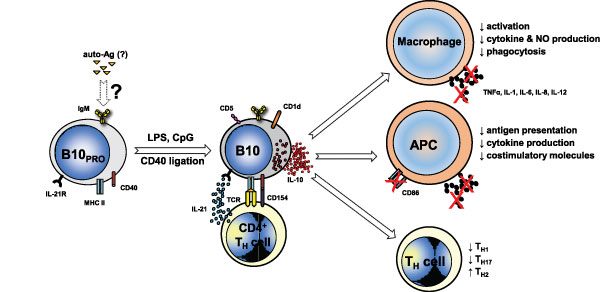
**B10 cell regulatory effects in autoimmune disease**. In this model, unidentified autoantigens (auto-Ags) drive early development of B10_PRO _cells. Following exposure to CD40 ligation and/or Toll-like receptor (TLR) ligands (lipopolysaccharide (LPS), CpG), B10_PRO _cells mature into B10 cells that can actively secrete IL-10 and regulate both innate and adaptive immune responses. IL-21R signaling along with major histocompatibility complex class II (MHC-II) and CD40 cognate interactions with CD4^+ ^T cells, although not needed for B10 cell development, are necessary for B10 cell effector functions and result in antigen-specific responses. B10 cells regulate macrophage function by decreasing their activation, phagocytosis and cytokine and nitric oxide (NO) production. In antigen-presenting cells (APCs), B10-cell-negative regulation of antigen presentation, expression of co-stimulatory molecules (such as CD86) and proinflammatory cytokine production limits T cell activation. In CD4^+ ^T helper (T_H_) cells, B10 cells skew responses towards a T_H2 _phenotype and away from T_H1 _and T_H17 _responses. The negative regulatory effects of B10 cells thereby limit inflammatory responses and subsequent tissue damage. Arrows with solid outline, known associations; arrows with dashed outline, speculated associations.

The most critical unsolved issue relates to the nature of antigenic stimuli driving B10 cell development. The identification of B10-cell BCR specificity is imperative since it will provide new insights into their early development. The autoreactive nature of mouse B10-cell BCRs [47] suggests that autoantigens may be driving early B10 cell development and that B10 cells may represent one of the ways enabling the immune system to peripherally tolerate autoantigens. B cells responding to autoantigens in an IL-10-dependent regulatory way can potentially limit inflammatory responses and limit autoimmune phenomena (see later section on B10 cell regulatory effects and Figure 2). Cell death products, by providing simultaneously both antigenic and nonantigenic stimuli, may represent one of the physiologic triggers for B10 cell development. The clearance of antigenic products of dying cells by noncomplement-fixing IgM polyreactive/autoreactive antibodies (such as those made by mouse B10 cells) in an IL-10-rich environment would be beneficial since it could potentially limit inflammatory responses to self-Ags. Additional unidentified antigenic and nonantigenic stimuli are probably involved in B10 cell development. The identification of such stimuli will provide additional insights ito B cell development that may prove invaluable for the future manipulation of B10 cells for treating autoimmune disease. Another important question is whether B10 cells enter the B cell memory pool during their development. This question is suggested by human studies demonstrating that B10_PRO _cells and B10 cells share phenotypic features with memory B cells (see later section on Human B10 cell phenotype).

### Mouse B10 cell phenotype

Although a variety of cell surface markers have been proposed [31,32], there is no known surface phenotype unique to B10 cells and, currently, the only way to identify these cells is functionally by intracellular IL-10 staining [35]. Only a small portion of B cells (that is, ~1 to 3% of splenic B cells in wildtype C57BL/6 mice) produce IL-10 following PMA and ionomycin stimulation, implying that not all B cells are competent to produce IL-10. Intracellular cytokine staining combined with flow cytometric phenotyping shows that mouse spleen B10 cells are enriched within the small CD1d^hi^CD5^+ ^B cell subset, where they represent 15 to 20% of the cells in C57BL/6 mice. This phenotypically unique CD1d^hi^CD5^+ ^subset shares overlapping cell surface markers with a variety of phenotypically defined B cell subsets such as CD5^+ ^B-1a B cells, CD1d^hi^CD23^−^IgM^hi^CD1d^hi ^marginal zone B cells, and CD1d^hi^CD23^+^IgM^hi^CD1d^hi ^T2 marginal zone precursor B cells, which all undoubtedly contain both B10_PRO _cells and B10 cells [23,26,30,50]. Mouse B10 cells are predominantly IgD^low^IgM^hi^, and <10% co-express IgG or IgA, but they can differentiate into antibody-secreting cells secreting polyreactive or Ag-specific IgM and IgG [47]. IL-10+ B cells were recently shown to be enriched in the TIM-1^+ ^compartment and TIM-1^+ ^B cells are enriched in the CD1d^hi^CD5^+ ^compartment [41]. However, IL-10^+ ^B cells are also present in the TIM-1^-- ^compartment and TIM-1^+ ^B cells are present in the non-CD1d^hi^CD5^+ ^compartment. Intracellular cytoplasmic IL-10 staining thereby remains the only current way to visualize the entire subset of IL-10-competent B cells. Nonetheless, the isolation of CD1d^hi^CD5^+ ^B cells or other phenotypically defined B cell subsets where B10 cells are enriched currently provides the best current means for isolating a viable B cell population that is significantly enriched for B10 cells and can be used for adoptive transfer experiments and functional studies in mice.

### Human B10 cell phenotype

The IL-10-producing B cell subset characterized in humans normally represents <1% of peripheral blood B cells [48]. Peripheral blood B10 cells and B10_PRO _cells are highly enriched in the CD24^hi^CD27^+ ^B cell subset, with approximately 60% also expressing CD38. Similar total numbers of IL-10^+ ^B cells have been described in the CD24^hi^CD38^hi ^and CD24^int^CD38^int ^B cell subsets [51]. A separate study showed that B10 cells did not fall within any of the previously defined B cell subsets, but they were enriched in the CD27^+ ^and the CD38^hi ^compartments [49]. Human B10 cells also highly express CD48 and CD148 [48]. CD48 is a B cell activation marker [52] and CD148 is considered a marker for human memory B cells [53]. CD27 expression is another well-characterized marker for memory B cells, although some memory B cells may be CD27^-- ^[54-56]. The CD27^+ ^B cell subset can also expand during the course of autoimmunity and has been proposed as a marker for disease activity [54,56]. The CD24^hi^CD148^+ ^phenotype of B10 cells and B10_PRO _cells may thereby indicate their selection into the memory B cell pool during development, or they may represent a distinct B cell subset that shares common cell surface markers with memory B cells. Consistent with a memory phenotype, the proliferative capacity of human blood B10 cells in response to mitogen stimulation is higher than that for other B cells [48], as is seen for mouse B10 cells [37]. Human transitional B cells are rare (2 to 3% of B cells) in adult human blood and are generally CD10^+^CD24^hi^CD38^hi ^cells that are also CD27-negative [55,56]; since CD10 expression is a well-accepted marker for most cells within the transitional B cell pool [57], its absence on B10 cells suggests that these cells are not recent emigrants from the bone marrow. In summary, human B10 cells share phenotypic characteristics with other previously defined B cell subsets, and, currently, there is no known surface phenotype unique to B10 cells.

## B10 cell regulatory effects

B10 cells exert a variety of IL-10-dependent regulatory effects potentially involved in autoimmune disease. The anti-inflammatory effects of IL-10 are mediated by multiple mechanisms involving both the innate and adaptive arms of the immune system. In innate cells, these mechanisms include downregulation of proinflammatory cytokine production [58] and decreased expression of MHC-II and co-stimulatory molecules [59] resulting in decreased T cell activation. B10 cells negatively regulate the ability of dendritic cells to present Ag [60]. In CD4^+ ^T cells, IL-10 suppresses T_H1 _[50] and enhances T_H2 _polarization [41,59]. B10 cells suppress IFNγ and TNFα responses *in vitro *[60] and INFγ responses *in vivo *[36] by Ag-specific CD4^+ ^T cells. Co-culture of mouse CD1d^hi^CD5^+ ^B cells with CFSE-labeled naive CD4^+ ^T cells suppresses T_H17 _cell differentiation [61] and IL-10 is known to suppress T_H17 _responses [62]. The suppression of T_H17 _responses by B10 cells *in vivo *was demonstrated recently [36]. IL-10 production by human B10 cells inhibits Ag-specific CD4^+^CD25^-- ^T cell proliferation [49] and regulates monocyte activation and cytokine production [48]*in vitro*.

A number of studies suggest that IL-10-producing B cells are important for the generation and/or maintenance of the regulatory T cell (T_REG_) pool [46,63-72]. However, a recent study [73] and our previously published data [23] do not support this view. The reason for this discrepancy is unclear but may be related to the different models of inflammation and conditions used to study the relationship of B10 cells and T_REGS_. These two studies suggesting that B10 cells are not involved in the generation and maintenance of the T_REG _pool are both in models of EAE [23,73]. In contrast, only one study suggests that B10 cells are important for the generation and/or maintenance of the T_REG _pool specifically in EAE [63]. The results of a different study clarify the picture in EAE further by showing that a subset of regulatory B cells control T_REG _numbers through IL-10-independent mechanisms [34]. Human B10 cell IL-10 production will therefore probably also have pleiotropic regulatory effects on the immune system, as occurs in mice. The potential regulatory effects of B10 cells in autoimmune disease limiting inflammatory responses and subsequent tissue damage are summarized in Figure 2.

## B10 cells in human autoimmune disease

Studies of B10 cells and human autoimmune disease are limited but of outmost importance since they provide valuable insights relevant to the potential future therapeutic application of B10 cells in humans. Peripheral blood B10 cells and B10_PRO _cells are present in patients with autoimmune diseases, including rheumatoid arthritis, systemic lupus erythematosus, primary Sjögren's syndrome, autoimmune bullous diseases, and multiple sclerosis. Interestingly, B10+B10_PRO _cell frequencies are expanded in some but not all cases, while mean B10+ B10_PRO _cell frequencies are significantly higher in patients with autoimmune disease compared with age-matched healthy controls [48]. A different study examined cytoplasmic IL-10 production by B cells from systemic lupus erythematosus patients and normal controls [74]. Blood mononuclear cells were cultured for 24 hours in the presence or absence of PMA, ionomycin, or LPS; significantly more systemic lupus erythematosus CD5^+ ^B cells produced cytoplasmic IL-10 than did controls. A different study also demonstrated spontaneous B cell IL-10 production that is higher in untreated rheumatoid arthritis, systemic sclerosis, and systemic lupus erythematosus patients than in controls [75].

By contrast, the concept of functional impairment of B10 cells in autoimmune disease was recently introduced by demonstrating functional impairment of CD24^hi^CD38^hi ^regulatory B cells in human systemic lupus erythematosus [51]. Cultures of peripheral blood mononuclear cells were stimulated with plate-bound anti-CD3 mAb for 72 hours, followed by the measurement of IFNγ and TNFα CD4^+ ^T cell responses. When CD24^hi^CD38^hi ^B cells were removed from the culture, higher frequencies of CD4^+^IFNγ^+ ^and CD4^+^TNFα^+ ^T cells were noted in healthy individuals but not in systemic lupus erythematosus patients; this effect was partially IL-10 dependent. In addition, CD24^hi^CD38^hi ^B cells isolated from the peripheral blood of systemic lupus erythematosus patients were refractory to CD40 ligation and produced less IL-10 compared with their healthy counterparts. The results of this study are rather intriguing but these findings need to be validated in view of the complexity of the culture system used and the non-uniformity of the CD24^hi^CD38^hi ^B cell subset with regards to its IL-10-dependent regulatory properties. In conclusion, B10 cells are present in the peripheral blood of autoimmune disease patients, where they appear to be expanded, whereas the functional capacity of human B10 cells in autoimmunity needs to be further defined.

## B10 cells in mouse models of autoimmune disease

The important regulatory effects of B10 cells *in vivo *and their therapeutic potential in autoimmunity have been demonstrated in a variety of mouse models of human autoimmune disease.

### Experimental autoimmune encephalomyelitis

EAE is an established model of multiple sclerosis induced by immunization with myelin peptides (such as myelin oligodendrocyte glycoprotein) leading to demyelination mediated by auto-Ag-specific CD4^+ ^T cells [76,77]. B cells were shown over a decade ago to have regulatory properties during the induction of EAE, with genetically B cell-deficient mice developing a severe nonremitting form of the disease [21]. However, these B cell regulatory effects were recently shown not to be IL-10 dependent [34]. Nonetheless, other studies highlight the importance of B cell-derived IL-10 in EAE. Specifically, EAE severity during the late phase of disease increases in B cell-deficient μMT mice that do not fully recover from their disease when compared with wildtype mice, and the adoptive transfer of wildtype B cells but not IL-10^--/-- ^B cells normalizes EAE severity in μMT mice [22]. Disease recovery is dependent on the presence of autoantigen-reactive B cells, and B cells isolated from mice with disease produced IL-10 in response to autoantigen stimulation. In the absence of Ag-specific B cell IL-10 production, the proinflammatory T_H1_-mediated immune responses persist and mice do not recover from the disease.

The EAE model demonstrates the complexity of regulatory mechanisms mediated by different cell subsets during different stages of the disease. When B cells from wildtype mice are depleted by CD20 mAb treatment 7 days before EAE induction, there is an increased influx or expansion of encephalitogenic T cells within the central nervous system and exacerbation of disease symptoms [23]. This effect is related to B10 cell depletion since similar effects are observed with selective B10 depletion by means of CD22 mAb [60]. The adoptive transfer of Ag-specific (myelin oligodendrocyte glycoprotein-sensitized) B10 cells into wildtype mice also reduces EAE initiation dramatically. The protective effect is IL-10 dependent since the adoptive transfer of CD1d^hi^CD5^+ ^B cells purified from IL-10^−*/*− ^mice does not affect EAE severity. B10 cell effector functions in EAE require IL-21 along with cognate interactions with CD4^+ ^T cells since the adoptive transfer of CD1d^hi^CD5^+ ^B cells into CD19^--/-- ^mice from IL-21R^--/--^, MHC-II^--/-- ^or CD40^--/-- ^mice prior to the induction of EAE does not alter disease course [36]. Once disease is established, adoptive transfer of B10 cells does not suppress ongoing EAE. B10 cells thereby appear to normally regulate acute autoimmune responses in EAE. In contrast to the role of B10 cells in early disease, T_REG _depletion enhances late-phase disease. Therefore, in EAE, depending on the stage of the disease, different regulatory mechanisms are involved in limiting inflammatory responses, with B10 cells regulating disease initiation and T_REGS _being involved predominantly in the regulation of late-phase disease.

### Inflammatory bowel disease

IL-10-producing B cells regulate intestinal inflammation in inflammatory bowel disease [26]. Early studies showed that B cells and their autoantibody products suppress colitis in T cell receptor alpha chain-deficient mice that spontaneously develop chronic colitis, while B cells are not required for disease initiation [78]. B cells with upregulated CD1d expression in the gut-associated lymphoid tissues of mice with intestinal inflammation were subsequently demonstrated to be regulatory [25]. This IL-10-producing B cell subset appears during chronic inflammation in T cell receptor alpha chain-deficient mice and suppresses the progression of intestinal inflammation by downregulating inflammatory cascades associated with IL-1 upregulation and signal transducer and activator of transcription 3 (*stat3*) activation rather than by altering polarized T_H _cell responses. The adoptive transfer of these mesenteric lymph node B cells also suppresses inflammatory bowel disease through a mechanism that correlates with an increase in T_REG _subsets [67]. Oral administration of dextran sulfate sodium solution to mice is widely used as a model of human ulcerative colitis. Dextran sulfate sodium-induced intestinal injury is more severe in CD19^--/-- ^mice (where B10 cells are absent) than in wildtype mice [79], and these inflammatory responses are negatively regulated by CD1d^hi^CD5^+ ^B cells producing IL-10. B10 cells therefore emerge during chronic inflammation in mouse models of inflammatory bowel disease, where they suppress the progression of inflammatory responses and ameliorate disease manifestations.

### Collagen-induced arthritis

CIA is a model for human rheumatoid arthritis that develops in susceptible mouse strains immunized with heterologous type II collagen emulsified in complete Freund's adjuvant [80,81]. CIA and rheumatoid arthritis share in common an association with a limited number of MHC-II haplotypes that determine disease susceptibility [82,83]. B cells are important for initiating inflammation and arthritis since mature B cell depletion significantly reduces disease severity prior to CIA induction but does not inhibit established disease [84]. Several studies on CIA demonstrate the negative regulatory effects and therapeutic potential of B10 cells.

Activation of arthritogenic splenocytes with Ag and agonistic anti-CD40 mAb induces a B cell population that produces high levels of IL-10 and low levels of IFNγ [85]. The adoptive transfer of these B cells into DBA/1--T cell receptor--β-Tg mice, immunized with bovine collagen (type II collagen) emulsified in complete Freund's adjuvant, inhibits T_H1 _responses, prevents arthritis development, and is effective in ameliorating established disease. The adoptive transfer of CD21^hi^CD23^+^IgM^+ ^B cells from DBA/1 mice in the remission phase prevents CIA and reduces disease severity through IL-10 secretion [86]; a significant but less dramatic therapeutic effect on CIA progression is seen when cells from naïve mice are adoptively transferred. In addition, the adoptive transfer of *ex vivo *expanded CD1d^hi^CD5^+ ^B cells in collagen-immunized mice delays arthritis onset and reduces disease severity, accompanied by a substantial reduction in the number of T_H17 _cells [61]. Co-culture of CD1d^hi^CD5^+ ^B cells with naive CD4^+ ^T cells suppresses T_H17 _cell differentiation *in vitro*, and co-culture of CD1d^hi^CD5^+ ^B cells with T_H17 _cells results in decreased proliferation responses *in vitro*. Furthermore, the adoptive transfer of T_H17 _cells triggers CIA in IL-17^--/-- ^DBA mice; however, when T_H17 _cells are co-transferred with CD1d^hi^CD5^+ ^B cells, the onset of CIA is significantly delayed. Finally, in a different study, administration of apoptotic thymocytes along with ovalbumin peptide and complete Freund's adjuvant to mice carrying an ovalbumin-specific rearranged T cell receptor transgene (DO11.10 mice) up to 1 month before the onset of CIA resulted in an increase in ovalbumin-specific IL-10 secretion and is protective for severe joint inflammation and bone destruction [46]. Activated spleen B cells responded directly to apoptotic cell treatment *in vitro *by increasing secretion of IL-10, and inhibition of IL-10 *in vivo *reversed the beneficial effects of apoptotic cell treatment [46].

### Systemic lupus erythematosus

B cell-negative regulatory effects are important in NZB/W mice, a spontaneous lupus model, since mature B cell depletion initiated in 4-week-old NZB/W F1 mice hastens disease onset, which parallels depletion of B10 cells [87]. B10 cells are phenotypically similar in NZB/W F1 and C57BL/6 mice, but are expanded significantly in young NZB/W F1 mice [87]. In wildtype NZB/W mice, the CD1d^hi^CD5^+^B220^+ ^B cell subset, which is enriched in B10 cells, is increased 2.5-fold during the disease course, whereas CD19^--/-- ^NZB/W mice lack this CD1d^hi^CD5^+ ^regulatory B cell subset [88]. Finally, the potential therapeutic effect of B10 cells in lupus is highlighted by the prolonged survival of CD19^--/-- ^NZB/W recipients following the adoptive transfer of splenic CD1d^hi^CD5^+ ^B cells from wildtype NZB/W mice [88]. Studies in the NZB/W spontaneous lupus model therefore suggest that B10 cells have protective and potentially therapeutic effects.

In the MRL.Fas(lpr) mouse lupus model, B cell-derived IL-10 does not regulate spontaneous autoimmunity [89]. B cell-specific deletion of IL-10 in MRL.Fas(lpr) mice indicates that B cell-derived IL-10 is ineffective in suppressing the spontaneous activation of self-reactive B cells and T cells during lupus. The severity of organ disease and survival rates in mice harboring IL-10-deficient B cells were unaltered. MRL.Fas(lpr) IL-10 reporter mice illustrate that B cells comprise only a small fraction of the pool of IL-10-competent cells. In contrast to previously published studies from our laboratory and elsewhere, putative regulatory B cell phenotypic subsets, such as CD1d^hi^CD5^+ ^and CD21^hi^CD23^hi ^B cells, were not enriched in IL-10 transcription. This observation suggests fundamental differences in the pathogenesis and immune dysregulation in the NZB/W lupus model compared with the MRL.Fas(lpr) model.

### Type 1 diabetes

Studies on B10 cells and mouse models of diabetes are limited to the nonobese diabetic (NOD) mouse, a spontaneous model of type 1 diabetes in which autoimmune destruction of the insulin-producing pancreatic β cells is primarily T cell mediated [90]. Although B cells clearly have a pathogenic role in disease initiation [91], B cells activated *in vitro *can maintain tolerance and transfer protection from type 1 diabetes in NOD mice [92,93]. The adoptive transfer of BCR-stimulated B cells into NOD mice starting at 5 to 6 weeks of age both delays the onset and reduces the incidence of type 1 diabetes, while treatment at 9 weeks of age delays disease onset. Protection from type 1 diabetes requires B cell IL-10 production since the adoptive transfer-activated NOD-IL-10^--/-- ^B cells do not confer protection from type 1 diabetes or the severe insulitis in NOD recipients. The therapeutic effect of adoptively transferred activated NOD B cells correlates with T_H2 _polarization. The limited data above suggest that B10 cells may be protective in preventing establishment of type 1 diabetes in NOD mice.

## Therapeutic potential of B10 cells

Harvesting the anti-inflammatory properties of B10 cells can provide a new approach to the treatment of autoimmunity. Manipulation of this subset for treating autoimmune disease is possible by either selective depletion of mature B cells while sparing B10/B10_PRO _cells or the selective expansion of B10 cells. Since there are no identified surface molecules specific for non-B10/B10_PRO _cells, it is currently impossible to selectively target and deplete mature B cells while sparing B10/B10_PRO _cells. B10 cell expansion appears to be a more viable approach since some of the stimuli driving their development have been identified. B10 cells can be expanded for therapeutic purposes either *in vivo *or *ex vivo*. Expansion of B10 cells *in vivo *by means of agonistic CD40 antibody has shown benefit in CIA [85]. However, expanding B10 cells *in vivo *carries additional risks since the currently identified stimuli driving B10 cell development are rather nonspecific and, if administered systemically, will trigger responses in a variety of immune cells. For example, the systemic administration of agonistic CD40 antibodies in humans has been associated with serious adverse effects such as cytokine release syndrome [94]. In summary, selective depletion of mature B cells while sparing B10/B10_PRO _cells is not currently possible, and *in vivo *B10 cell expansion by nonspecific agents such as agonistic CD40 antibody is potentially associated with serious off-target effects.

Expanding B10 cells *ex vivo *appears more preferable than *in vivo *B10 cell expansion by nonspecific agents because it offers a potential therapy without the risk of undesirable nonspecific off-target effects. However, *ex vivo *B10 cell expansion introduces new challenges related to the method of expansion, to the magnitude of expansion and to the time it takes to generate B10 numbers that will be sufficient for therapeutic use. The method of *ex vivo *B10 cell expansion can be the source of safety concerns when it comes to human applications. Large numbers of regulatory B cells have been successfully generated in mice by means of genetic manipulation of immature B cells through lentiviral transfection [95]. These cells were effective in treating EAE. However, although this method can efficiently generate large numbers of regulatory B cells *ex vivo*, concerns remain about administering infusions of lentivirus-infected B cells to humans (with retroviral and infectious potential). Safety concerns thereby limit the use of infectious agents in manipulating human cells, which could render this approach inappropriate for use in humans.

The magnitude of *ex vivo *B10 cell expansion is very important since the number of cells infused during adoptive transfer experiments is critical. In humans, the most convenient potential source of B10/B10_PRO _cells prior to *ex vivo *expansion is obviously peripheral blood. Since B10/B10_PRO _cells are rare in peripheral blood and there are limitations on the volume that can be drawn at any given time, a method of expanding B10 cells by several million-fold is needed. Furthermore, since this method will be used for treatment of active disease, the time it will take to expand these cells *ex vivo *is also of great significance; ideally, this process should not take more than 1 or 2 weeks. There is accumulating hope that such an approach will soon be available for human cells since mouse B10 cell *ex vivo *expansion can be accomplished within 9 days by means of combined CD154, B-lymphocyte stimulator, IL-4 and IL-21 stimulation [36]. After the 9-day culture period, B10 cell numbers are increased 4,000,000-fold, with 38% of the B cells actively producing IL-10. Fluorescence-activated cell sorting based on CD5 expression increases the B10 cell purity to75%, thus providing not only large numbers of B10 cells but also a B cell population predominantly consisting of B10 cells. These *ex vivo *expanded B10 cells are very effective in limiting inflammatory responses in EAE. This approach appears promising since it provides an effective way of generating large numbers of B10 cells without the use of infectious agents. The development of a similar system for expanding human B10 cells is of outmost importance.

## Conclusion

The phenotypic and functional characterization of B10 cells is an important advance for the regulatory B cell field. Numerous additional functionally defined subsets of regulatory B cells will probably be identified in the future. B10 cells share phenotypic markers with a variety of previously defined subsets, but their only unique phenotypic marker is intracellular IL-10 production. Although certain transcription factors are involved at different points in B10 cell development, there is currently no transcription factor signature unique to B10 cells. BCR-related signals are most critical in B10 cell development and the finding of B10-cell BCR auto-reactivity suggests that autoantigens may be of particular importance. The recent discovery of an *in vitro *method to efficiently expand mouse B10 cells provides an invaluable tool for studying the basic biology of B10 cells as well as manipulating them for therapeutic purposes. The development of a similar method for human cells will open new opportunities for studying the basic biology of human B10 cells and a promising novel approach in treating human autoimmune disease, potentially without undesirable off-target effects.

## Abbreviations

Ag: antigen; B10 cells: IL-10-producing regulatory B cells; BCR: B cell receptor; CIA: collagen-induced arthritis; EAE: experimental autoimmune encephalomyelitis; IFN: interferon; IL: interleukin; LPS: lipolysaccharide; mAb: monoclonal antibody; MHC: major histocompatibility complex; MyD88: myeloid differentiation primary response gene 88; NOD: nonobese diabetic; PMA: phorbol-12-myristate-13-acetate; T_H_: T-helper; TIM-1: T cell immunoglobulin domain and mucin domain protein 1; TLR: Toll-like receptor; TNF: tumor necrosis factor; T_REG_: regulatory T cell.

## Competing interests

The authors declare that they have no competing interests.

## Declarations

This article has been published as part of *Arthritis Research & Therapy *Volume 15 Supplement 1, 2013: B cells in autoimmune diseases: Part 2. The supplement was proposed by the journal and content was developed in consultation with the Editors-in-Chief. Articles have been independently prepared by the authors and have undergone the journal's standard peer review process. Publication of the supplement was supported by Medimmune.
